# Evaluation of Computed Tomography Scoring Systems in the Prediction of Short-Term Mortality in Traumatic Brain Injury Patients from a Low- to Middle-Income Country

**DOI:** 10.1089/neur.2021.0067

**Published:** 2022-04-14

**Authors:** Matheus Rodrigues de Souza, Mayra Aparecida Côrtes, Gustavo Carlos Lucena da Silva, Davi Jorge Fontoura Solla, Eryanne Garcia Marques, Wellithon Luz Oliveira Junior, Caroline Ferreira Fagundes, Manoel Jacobsen Teixeira, Robson Luis Oliveira de Amorim, Andres M. Rubiano, Angelos G. Kolias, Wellingson Silva Paiva

**Affiliations:** ^1^ Department of Medicine, Mato Grosso State University, Cáceres, Brazil.; ^2^ Department of Neurology–Division of Neurosurgery, University of São Paulo, São Paulo, São Paulo, Brazil.; ^3^ NIHR Global Health Research Group on Neurotrauma, Addenbrooke's Hospital, University of Cambridge, Cambridge, United Kingdom.; ^4^ Department of Neurosurgery–Neuroscience Institute, Neurotrauma Group, El Bosque University, Bogotá, Colombia.; ^5^ Department of Clinical Neuroscience–Division of Neurosurgery, Addenbrooke's Hospital, University of Cambridge, Cambridge, United Kingdom.

**Keywords:** CT scoring, prognostic models, traumatic brain injury

## Abstract

The present study aims to evaluate the accuracy of the prognostic discrimination and prediction of the short-term mortality of the Marshall computed tomography (CT) classification and Rotterdam and Helsinki CT scores in a cohort of TBI patients from a low- to middle-income country. This is a *post hoc* analysis of a previously conducted prospective cohort study conducted in a university-associated, tertiary-level hospital that serves a population of >12 million in Brazil. Marshall CT class, Rotterdam and Helsinki scores, and their components were evaluated in the prediction of 14-day and in-hospital mortality using Nagelkerk's pseudo-*R*^2^ and area under the receiver operating characteristic curve. Multi-variate regression was performed using known outcome predictors (age, Glasgow Coma Scale, pupil response, hypoxia, hypotension, and hemoglobin values) to evaluate the increase in variance explained when adding each of the CT classification systems. Four hundred forty-seven patients were included. Mean age of the patient cohort was 40 (standard deviation, 17.83) years, and 85.5% were male. Marshall CT class was the least accurate model, showing pseudo-*R*^2^ values equal to 0.122 for 14-day mortality and 0.057 for in-hospital mortality, whereas Rotterdam CT scores were 0.245 and 0.194 and Helsinki CT scores were 0.264 and 0.229. The AUC confirms the best prediction of the Rotterdam and Helsinki CT scores regarding the Marshall CT class, which presented greater discriminative ability. When associated with known outcome predictors, Marshall CT class and Rotterdam and Helsinki CT scores showed an increase in the explained variance of 2%, 13.4%, and 21.6%, respectively. In this study, Rotterdam and Helsinki scores were more accurate models in predicting short-term mortality. The study denotes a contribution to the process of external validation of the scores and may collaborate with the best risk stratification for patients with this important pathology.

## Introduction

Traumatic brain injury (TBI) is the leading cause of death and neurological disability worldwide, especially in low- to middle-income countries (LMICs).^1,2^ In Brazil, an incidence rate of 65.7 hospital admissions per 100,000 inhabitants per year is estimated, with a mortality rate of 5.1 per 100,000, leading to economic losses of >$70,960,000 USD.^3^

Accurate prognostic information, especially in the context of admitting patients with TBI, plays an important role in clinical decision making, resource allocation, and communication between doctors and family members. Computerized tomography (CT) is an objective means of quantifying parenchymal and bone lesions in patients suffering from TBIs, especially in the acute phase.^4–6^ Protocols and guidelines on the prognosis of TBI include CT as a predictor based on evidence class I.^7^ The information provided by the admission CT allows, in addition to the diagnostic screening for potential lesions that require a surgical approach, for obtaining important prognostic information. Outcome prediction models can help prioritize resources in emergency care and also have the potential to improve clinical TBI research by providing a baseline for risk stratification.^8,9^

To systematize and stratify TBI patients based on CT imaging characteristics, several classification systems have been proposed; the most popular used in practice are Marshall CT classification and Rotterdam and Helsinki CT scores.

Marshall CT classification was proposed in 1991 and evaluates three main findings: the status of the perimesencephalic cisterns; midline structure deviation; and focal lesions that depend on the volume of the lesion.^10^ Although its components have shown an association with clinical outcomes, Marshall class was not originally proposed as a prognostic tool, and its cut-off points were defined according to mortality risk in a population of patients managed with protocols of care from the early 1980s, where aggressive surgical management for high intracranial pressure (ICP) was not a common approach.^11^

In 2005, the Rotterdam score was proposed, which reassessed the components of Marshall's classification and added traumatic subarachnoid hemorrhage (tSAH), intraventricular hemorrhage (IVH), and epidural hematomas, creating an ordinal score criterion. This score was designed based on IMPACT (International Mission for Prognosis and Clinical Trials in TBI) study findings, being a secondary analysis of multi-centric studies from the 1980s and early 1990s, where aggressive surgical management for high ICP was also not a common option.^12^ Recently, the Helsinki score was proposed; it subdivides the evaluation into four criteria: the type of mass injury; the size of the injury; whether there is the presence of IVH; and the suprasellar cisterns.^13^ A recent study showed that the Helsinki score proved to be a more accurate predictor of outcomes in TBI patients because it has been developed with the findings of the managing of a cohort of patients with more-recent protocols.^9^ However, studies on prognostic models based on tomography findings are scarce in LMICs.

These countries have the highest rate of neurotrauma; however, most of the scientific articles published in journals originate from high-income countries.^14–16^ Moreover, the tomography scoring systems were validated in different epidemiological contexts than those presented by LMICs. Thus, the primary objective of the present study is to evaluate the accuracy of the prognostic discrimination and prediction of the short-term mortality of the Marshall CT classification and Rotterdam and Helsinki CT scores in a cohort of TBI patients from an LMIC. Our secondary objectives were to evaluate the individual components of each score and determine the prognostic value of these scoring systems associated with the variables present in the IMPACT prognosis calculator.

## Methods

### Study design

This is a *post hoc* analysis of a previously conducted prospective cohort study. The study adhered to the principles of the Transparent Reporting of a multi-variable prediction model for Individual Prognosis or Diagnosis (TRIPOD): the TRIPOD statement.^17^

### Patients and population

The study was conducted at the Clinics Hospital of the University of São Paulo, a tertiary-level hospital located in the largest city of Brazil, serving a population of >12 million. This analysis included consecutive patients admitted to the emergency department from January 2012 to December 2015. Our registry includes patients with TBI, defined as any patient requiring admission to an intensive care trauma unit and referred to the neurosurgery team. Pre-hospital data were collected through the analysis of the clinical chart of the rescue team. We only included patients >14 years of age and patients diagnosed with an intracranial abnormality on initial head CT scan. We excluded patients with penetrating TBI, as well as those with a Glasgow Coma Scale (GCS) of 15 and the ones without an intracranial lesion on the CT scan. In our institution, any patient with intracranial abnormalities is eligible to be transferred to the intensive care unit (ICU), which is subject to the availability of a bed. Therapeutic planning followed recommendations provided by Advanced Trauma Life Support, as well as guidelines provided by the Brain Trauma Foundation, whenever possible.

This study followed the principles of the Declaration of Helsinki and was approved by the Research Ethics Committee of the University of São Paulo School of Medicine (Registry: 46831315.3.0000.0068). The patients/participants provided their written informed consent to participate, and none of them are identified in this research.

### Variables of interest

Variables were selected based on the predictive models previously described in the literature as well as the information needed to calculate the scores obtained from the admission CT. The clinical data evaluated were sex, age, GCS, assessment of pupil response, and presence of hypoxia, hypotension, and hemoglobin values also referring to the admission to the service.

### Definition of radiological parameters

Regarding the initial CT findings, the following were evaluated: the presence of midline deviation >5 mm; cerebral hernia detected at CT (defined as an efface of the third ventricle or the basal cisterns); epidural hemorrhage; subdural hemorrhage; and intraparenchymal hemorrhage.

Marshall's classification was defined as suggested by previous studies, in which grade V (“lesion with evacuated mass”) and grade VI (“lesion with non-evacuated mass”) were grouped.^12,13^ The Rotterdam score was classified in increasing levels of severity, as suggested by the authors who validated this model,^12^ in the same way as the Helsinki score.^13^ The parameters of each rating model are shown in Table 1.

**Table 1.** Scoring Systems for the Assessment of CT in Patients with TBI

CT classification	Classification or component	Description
Marshall CT classification	Diffuse Injury Grade I	No visible intracranial pathology
	Diffuse Injury Grade II	Midline shift of 0–5 mm, basal cisterns remain visible, no high- or mixed-density lesions >25 cm^3^
	Diffuse Injury Grade III	Midline shift of 0–5 mm, basal cisterns compressed or completely effaced, no high- or mixed-density lesions >25 cm^3^
	Diffuse Injury Grade IV	Midline shift >5 mm, no high- or mixed-density lesions >25 cm^3^
	Diffuse Injury Grade V + IV	High- or mixed-density lesions >25 cm^3^
Rotterdam CT score	Basal cisterns	0: normal, 1: compressed, 2: absent
	Midline shift	0: no shift or ≤5 mm,1: shift >5 mm
	Epidural mass lesion	0: present, 1: absent
	Intraventricular hemorrahage or tSAH	0: present, 1: absent
	**Score**	**Range: 1– 6**
Helsinki CT score	Mass lesion type	Subdural hematoma: 2, intracerebral hematoma: 2, epidural hematoma: −3
	Mass lesion size	Hematoma volume >25 cm^3^
	Intraventricular hemorrahage	Present: 3
	Suprasellar cisterns	Normal: 0, compressed: 1, absent: 5
	**Score**	**Range: −3 to 14**

CT, computerized tomography; TBI, traumatic brain injury; tSAH, traumatic subarachnoid hemorrhage.

### Outcome and follow-up

Patients were followed throughout their hospital stay. Predictive value of the scales was evaluated for the primary outcome of mortality in 14 days, given a recent work that notes this as a useful point to evaluate short-term mortality in TBI^18^ and for the secondary outcome of in-hospital mortality.

### Statistical analysis

Categorical variables are presented using relative and absolute frequencies. Continuous data, when normally distributed, are presented as mean and standard deviation, or otherwise by median and interquartile. A *t*-test was used to compare numerical variables; for categorical variables, the chi-squared test was used.

Marshall's classification as well as the Rotterdam and Helsinki scores were treated as categorical variables. Nagelkerke's pseudo-*R*^2^ and the area under the receiver operating characteristic (ROC) curve (AUC) were used to evaluate the accuracy and discriminative ability of the models, used for their comparison. Nagelkerke's pseudo-*R*^2^ is a measure of the proportion of variability in the outcome that is explained by the logistic regression model. The AUC varies from 0.5 (no discrimination) to 1.0 (perfect discrimination). It is accepted that AUCs <0.6 reflect little discrimination; 0.60–0.75 possibly useful discrimination; and values >0.75 a useful discrimination.^19^ The non-parametric model of DeLong and colleagues was used to compare the discriminative ability of each score.^20^ Overall performance (how well the model predicts the likelihood of an outcome in an individual patient) was assessed using the Brier Score, which ranges from 0 to 1.^21^ A lower score indicates better model calibration.

Finally, multi-variate regression was performed using the data from the IMPACT Calculator^11^ to evaluate the increase in variance explained when adding each of the CT classification systems. Regarding the data evaluated by IMPACT, only blood glucose was not included, given that it was not available in the patient database. IMPACT data were added in regression by the insert model, which forces the entry of all variables. In addition, the scores were added in a second block to evaluate its increment in the standard model.

All tests were bilateral, and the value of *p* < 0.05 was considered statistically significant. Statistical analysis was conducted by the Software Statistical Package for Social Sciences (IBM SPSS Statistics for Windows, version 25.0; IBM, Armonk, NY), and the study of the ROC curves was conducted by MedCalc software (version 19.4.1; MedCalc Software Ltd., Ostend, Belgium).

## Results

### Patient characteristics

A total of 447 patients were included in the study. Mean age was 40 years (standard deviation, 17.83) and ranged from 14 to 99. There was a predominance of males, 85.5% (*n* = 382), which denotes a ratio of 5.87 men for each woman. Regarding the outcome, in 14 days, 22.8% (*n* = 102) of patients have evolved to death, and during the entire hospital stay, 33.8% (*n* = 151) did not survive. Table 2 presents in detail the general characteristics of the sample according to 14-day mortality outcome.

**Table 2.** Patient Characteristics for 14-Day Mortality Outcome

Variable	No. of patients (%)	Alive	Death	*p* value
Age, years				<0.001
>18	16 (3.6)	10 (03.4)	06 (4)	
18–29	120 (26.8)	102 (34.5)	18 (11.9)	
30–39	103 (23)	73 (24.7)	30 (19.9)	
40–49	75 (16.8)	48 (16.2)	27 (17.9)	
<50	133 (29.8)	63 (21.3)	70 (46.4)	
Sex				0.003
Male	382 (85.5)	260 (87.8)	122 (80.8)	
Female	65 (14.5)	36 (12.2)	29 (19.2)	
TBI class				0.002
Mild: GCS 13–15	96 (21.5)	80 (27)	16 (10.6)	
Moderate: GCS 9–12	64 (14.3)	46 (15.5)	18 (11.9)	
Severe: GSC 3–8	287 (64.2)	170 (57.4)	117 (77.5)	
Mechanism of injury				<0.001
RTI	262 (58.6)	184 (62.2)	78 (51.7)	
Fall	136 (30.4)	77 (26.0)	59 (39.1)	
Other	49 (11)	35 (11.8)	14 (9.3)	
Pupil responsiveness				<0.001
Responsive	365 (81.7)	272 (91.9)	93 (61.6)	
Unilateral unresponsive	56 (12.5)	20 (6.8)	36 (23.8)	
Bilateral unresponsive	26 (5.8)	04 (1.4)	22 (14.6)	
Marshall CT class				<0.001
Diffuse Injury I	18 (4.0)	13 (4.4)	05 (3.3)	
Diffuse Injury II	193 (43.2)	119 (40.2)	74 (49)	
Diffuse Injury III	37 (8.3)	22 (7.4)	15 (9.9)	
Diffuse Injury IV	20 (4.5)	13 (4.4)	07 (4.6)	
Diffuse Injury V	175 (39.1)	127 (42.9)	48 (31.8)	
Diffuse Injury VI	04 (0.9)	02 (0.7)	02 (1.3)	
Rotterdam CT score^a^				<0.001
1	09 (2)	09 (100)	—	
2	68 (15.2)	64 (18.6)	04 (3.9)	
3	205 (45.9)	181 (52.2)	24 (23.5)	
4	63 (14.1)	50 (11.6)	24 (23.5)	
5	57 (12.8)	31 (9)	26 (25.5	
6	45 (10.1)	20 (5.8)	25 (24.5)	
Helsinki CT score^a^				<0.001
	03 (0/5)	02 (0/4)	05 (3/9)	
Hypoxia				0.002
Present	52 (11.6)	33 (9.6)	19 (18.6)	
Missing	192 (43)	161 (46.7)	31 (30.4)	
Unknown	203 (45.4)	151 (43.8)	52 (51)	
Hypotension				0.001
Present	54 (12.1)	32 (9.3)	22 (21.6)	
Missing	350 (78.3)	281 (81.4)	69 (67.6)	
Unknown	43 (9.6)	32 (9.3)	11 (10.8)	
Hemglobin (g/dL)^a^				0.088
	11.6 (10/13)	11.8 (10.17/13.12)	11.2 (9.95/12.45)	
14-day mortality	**—**	345 (77.2)	102 (22.8)	—
In-hospital mortality	**—**	151 (33.8)	151 (33.8)	—

^a^ Median (interquartile range). All other variables: number (%).

GCS, Glasgow Coma Scale; MOI, mechanism of injury; RTI, road traffic injury.

### Outcome prediction of computed tomography scores

**FIG. 1.**
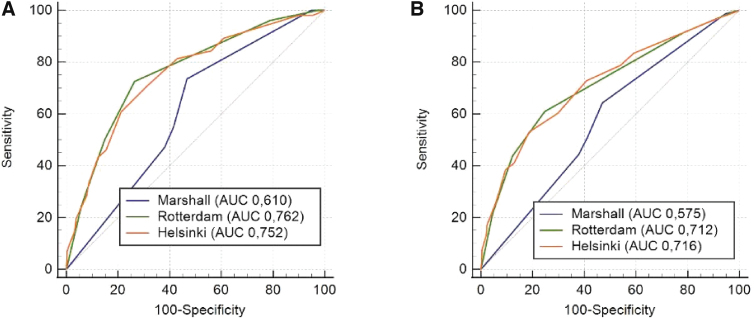
Receiver operating characteristic curves for prediction of (**A**) 14-day mortality and (**B**) in-hospital mortality. AUC, area under the receiver operating characteristic curve.

Rotterdam and Helsinki scores showed a better performance regarding Marshall's classification, both in predicting 14-day mortality and in-hospital mortality. Marshall CT class was the least accurate model, showing pseudo-*R*^2^ values equal to 0.122 for 14-day mortality and 0.057 for in-hospital mortality, whereas Rotterdam CT scores were 0.245 and 0.194 and Helsinki CT scores were 0.264 and 0.229 (Table 3). The AUC, demonstrated in Figure 1, confirms the best prediction of the Rotterdam and Helsinki CT scores regarding the Marshall CT class, which presented greater discriminative ability. These two models also presented better calibration, demonstrated by higher Brier Score values (Table 3).

**Table 3.** Variation Explained, Discriminative Ability, and Calibration of All Scores for Predicting Outcome in TBI

	Pseudo-*R*^2^	AUC (95% CI)	Brier Score
14-day mortality			
Marshall CT class	0.122	0.610 (0.553–0.668)	0.172
Rotterdam CT score	0.245	0.762 (0.709–0.815)	0.147
Helsinki CT score	0.264	0.752 (0.698–0.807)	0.149
In-hospital mortality			
Marshall CT class	0.057	0.575 (0.520–0.629)	0.183
Rotterdam CT score	0.194	0.712 (0.659–0.764)	0.158
Helsinki CT score	0.229	0.716 (0.664–0.767)	0.161

TBI, traumatic brain injury; CT, computerized tomography; AUC, area under the curve; CI, confidence interval.

Comparison of AUCs by the model of DeLong and colleagues demonstrated a statistical difference in the discriminative ability of both Rotterdam and Helsinki scores when compared with Marshall's classification. The discretely higher Rotterdam CT AUC value was not statistically significant when compared to the Helsinki CT, and *p* values for the comparison are presented in Supplementary Table S1.

### Evaluation of individual components of each score

The base cisterns component, present in the Rotterdam score, was the one that was most associated with the 14-day mortality outcome, with a Nagalkerke pseudo-*R*^2^ equal to 0.215. As for the Helsinki score components, evaluation of the suprasellar cisterns showed higher values of the Nagalkerke pseudo-*R*^2^ (0.176). The description of the analysis for each scoring item is presented in Supplementary Table S2.

### Outcome prediction of computed tomography scores associated with clinical data

Initially, a univariate logistic regression was performed for each item that makes up the IMPACT Calculator (except for the glucose values that were not available in the sample studied); the results are presented as base components in Supplementary Table S2.

Traditionally, only those variables whose *p* value is <0.002 by the univariate analysis are included in the multi-variate analysis. Hypoxia and hemoglobin values did not meet this criterion; however, these were included in this analysis because they are predictors of known results and are firmly described in the literature for TBI patients.^11,18,22^

All CT scores showed a significant increase in mortality prediction at 14 days when associated with the standard model. The addition of the Marshall CT class, although statistically significant, allowed for an increase of only 2% of the additional explained variance about the model without this predictor. Helsinki score, the one with the highest percentage, explained additional variance (21.6%) when compared to the standard model without this predictor. The analysis of discriminative ability by the AUC corroborates what was demonstrated by Nagelkerke's pseudo-*R*^2^. The results of the multi-variate analysis are summarized in Table 4.

**Table 4.** CT Models in Multi-Variable Analysis Together with Available IMPACT Variables

Model	Omnibus test	Nagelkerke's Pseudo-*R*^2^	AUC (CI 95%)	Brier Score
Base model	—	0.354	0.802 (0.723–0.882)	0.121
Base model + Marshall CT	0.038	0.376	0.812 (0.735–0.890)	0.120
Base model + Rotterdam CT	<0.001	0.492	0.880 (0.818–0.941)	0.114
Base model + Helsinki CT	<0.001	0.570	0.898 (0.844–0.953)	0.117

The base model consists of: age, better motor response on the Glasgow Coma Scale, pupillary response, hypoxia, and hypotension and hemoglobin levels. The Omnibus test *p* values describe whether the included score significantly added independent information to the standard model.

CT, computerized tomography; IMPACT, International Mission for Prognosis and Clinical Trials in TBI; AUC, area under the curve; CI, confidence interval.

## Discussion

The present study analyzed TBI victims in an LMIC to assess the accuracy of CT scores in predicting short-term mortality. Rotterdam and Helsinki scores were more accurate compared with the Marshall classification. When evaluating the individual components used in each score, the base cisterns item present in the Rotterdam score was the one most associated with 14-day mortality outcome. So far, this is the first study to evaluate the performance of CT scores for TBI patients in Brazil and one of the few to be done in an LMIC. Although these countries have the highest rates of disease because of trauma, it is observed that most of the knowledge produced on the subject in the literature comes from high-income countries.^15,16^

TBI is a devastating global health issue, affecting an estimated 69 million persons per year. Its impact, however, is not homogeneous among high and low Human Development Index countries, which have faced different recent transformations on TBI epidemiology.^23^ Low-income countries, with less resource availability, observe a TBI incidence increase partially attributable to an expansion in the number of motor vehicles. On the other side, populational aging poses new challenges to high-income countries, along with the proportional increment on standing height falls and victims' basal frailty.^24^ Brazil is situated at the low- to middle-income stratum and deals simultaneously with both sides of the aforementioned spectrum of transformations. It is estimated that >1 million Brazilians are victims of TBI annually, of which 20–30% are classified as moderate or severe.^25^ According to data from the Hospital Information System of the Brazilian Unified Health System Informatics Department, there was a >10% increase in the number of hospitalizations attributable to TBI over the past 10 years—currently, >100.000 per year.^2,3,26,27^

Accurate prognostic information is of utmost importance to patients as well as in determining the appropriate life-threatening conduct to which patients are exposed.^19^ In the context of TBI, the adoption of effective measures and behaviors has significant potential to modify patient outcome(s).^28,29^ The use of simplified prognostic models that are easily applicable in the clinical setting and incorporate the main points related to the outcome is extremely important.^22,30^

Therefore, the results of the study presented, which validate the risk-stratification models from the tomography data in a different epidemiological context from the one they were originally proposed, can stimulate their routine use and, from that, help in the decision-making process, in the allocation of resources, as well as in facilitating risk communication in a readily accessible way for physicians, other health professionals, patients, and caregivers.

The superiority of the Rotterdam and Helsinki scores over Marshall's classification, which is still widely used today, has been demonstrated. The pseudo-explained variances of the new scores are up to twice as large as Marshall's classification. This classification was built in 1991 from the data of 746 patients with severe TBI; however, throughout the years of its use, several studies have shown its low predictive value.^9,13,31,32^

In our study, the predictive value showed a pseudo-*R*^2^ of 0.122 and an AUC of 0.610 for 14-day mortality. The study by Raj and colleagues^13^ described the predictive value of this classification for 6-month mortality and found even lower values of pseudo-*R*^2^ (0.087). Recently, a multi-center study, when evaluating the performance of CT scores for functional outcome, found a low predictive and discriminative ability of Marshall's classification (pseudo-*R*^2^, 0.02; AUC, 0.580).^9^

Marshall's classification was not originally constructed for outcome prediction, given that it is not an ordinal score, and even the authors themselves recognize that grade IV is worse than grades V and VI.^10^ Moreover, this classification does not take into account important brain lesions, such as tSAH, and does not distinguish between subdural and epidural hematomas, which are known to have different prognoses.^33–36^ Thus, the present study supports something that had been previously demonstrated; Marshall's classification does not present itself as a good predictor of acute clinical prognosis, medium term, and for functional outcomes.^12,31,37^

Mass and colleagues^12^ proposed reorganizing Marshall's classification, distinguishing in a more detailed way the mass lesions, evaluating the basal cisterns, and adding the evaluation of the presence of traumatic or intraventricular subarachnoid hemorrhage, creating the Rotterdam score. The AUC obtained in that study for mortality at 6 months was like ours for more short-term mortality (0.748 vs. 0.762).

Like the original study, we found that the evaluation of the base cisterns is the component that presents the highest predictive value within the scale. Other authors have already demonstrated the value of the state of the base cisterns; the compression or obliteration of these indicate a deformation in the structures of the brain stem, responsible for several vegetative functions.^38,39^ Its compression is associated with respiratory failure, coma, and death. Otherwise, the compression of base cisterns may indicate a reduction in blood flow in the great vessels, especially in the posterior cerebral arteries territory at the base of the skull, and contribute to a worse outcome attributable to the phenomenon of ischemia in brain stem structures.^36^

Another important component of the scores that were not present in Marshall's classification was the presence of tSAH, which proved to be an independent predictor of mortality in our study from the univariate analysis (Nagelkerke's pseudo-*R*^2^, 0.048). Diffuse bleeding from ruptured subarachnoid vessels in TBI is a well-described predictor in the literature.^9,40,41^ Similar to what happens in aneurysm rupture, traumatic induce vasospasm and cerebral ischemia, which can trigger inflammatory and neurotoxic processes, which contribute to the deterioration of the patients' outcome.^42,43^

Marshall's and Rotterdam's scores are based on data of patients managed in the '80s and early '90s, they overestimate mortality in patients managed with more recent protocols, especially in patients that underwent decompression procedures. Considering the particularities of epidemiological characteristics of TBI in LMICs^23,26^ and that neurosurgeons dealing with neurotrauma in hospitals with limited neuromonitoring resources in the ICU use damage control almost daily,^44,45^ the present study stands out for evaluating the prediction of mortality from these scores in a recent database.

Helsinki score, developed with the findings of the management of a cohort of patients with more recent protocols, assigned different scores for each type of brain lesion (subdural, intraparenchymal, and epidural hematoma) and evaluated the size of the lesion and the status of the suprasellar cisterns. In the original study, it showed a better performance when compared to the others. The model proposed by the authors showed good predictive (Nagelkerke's pseudo-*R*^2^, 0.203) and discriminative ability (AUC, 0.744) for 6-month mortality.

We point out that in the present study both the predictive (Nagelkerke's pseudo-*R*^2^: 0.264) and the discriminative ability (AUC, 0.752) were greater than those found in the original study. Notwithstanding, it should be noted that the performance of the models for short-term mortality (14-day mortality) was evaluated, and the results were numerically higher because, over a longer period of hospitalization and clinical follow-up of patients, other variables started to contribute to the clinical outcome. The reduction of predictive and discriminatory capacity of all scales, when comparing the 14-day mortality outcome with in-hospital mortality, corroborates with the assumption of the additional contribution of other factors external to TBI.

The tomography findings are not interpreted separately in medical care; some parameters of patient admission play an important prognostic role. In this sense, the use of prognostic calculators, such as IMPACT or CRASH (Corticosteroid Randomisation After Significant Head Injury), provides predictions that can support clinical practice and the conduct of research.^11^ In this study, a significant increase in the addition of the Rotterdam and Helsinki scores to the accuracy of the clinical and laboratory data present in the IMPACT Prognosis Calculator was demonstrated, with emphasis on the Helsinki score, which added 22% to the variance explained in the isolated data of the standard model (Nagelkerke's pseudo-*R*^2^ of the standard model: 0.354 vs. 0.570 when associated with the Helsinki score).

Several studies that evaluate the prognosis based on CT findings include only patients classified as moderate and severe TBI.^10,13,30,31,46^ This classification comes from the GCS score, which, despite presenting a well-characterized discriminatory capacity in the literature, is subject to the influence of several other factors, such as the use of sedatives, drugs, alcoholic libation, and related to the subjective nature of its evaluation.^46–49^ This study included patients who were admitted to an ICU environment, regardless of their GCS score; thus, it is understood that this cohort of patients represents a clinically valid, useful group for the initially proposed objectives.

### Study limitations

Some limitations of the study should be discussed. Despite the significant number of patients, the study was restricted to a single center, which may limit the generalization of findings. However, the present study denotes a relevant contribution to the literature on the subject, from which it becomes important that other authors and different centers around the world evaluate the accuracy and discriminatory capacity of CT classification models for trauma patients, contributing to their external validation process.

Emphasis is also placed on the limitation of not providing data on the functional and long-term outcome of the population studied, and it is therefore suggested that authors of other LMICs evaluate the Glasgow Outcome Scale as an outcome in future studies. However, it should be noted that the difficulty of long-term follow-up of TBI patients is not restricted to this study and has been previously reported in the literature.^48,50,51^

## Conclusion

The present study was the first to evaluate the predictive value and discriminative ability of different classification systems and CT scores of TBI patients in the Brazilian population, an LMIC, where there is a high incidence of neurotrauma-related disease. It was demonstrated, from Nagelkerke's pseudo-*R*^2^ values and the AUC, that the Rotterdam and Helsinki scores are more accurate models to predict short-term mortality. In parallel, it was demonstrated that evaluation of the base and suprasellar cisterns are the parameters of the scores most associated with the assessed outcome. Moreover, the important increase in variance explained by adding the Helsinki score to the IMPACT Prognosis Calculator data was detailed. The study denotes a contribution to the process of external validation of the scores and may collaborate with the best risk stratification for patients with this important pathology.

## Data Availability Statement

The data sets generated for this study are available on request to the corresponding author.

## Supplementary Material

Supplemental data

Supplemental data

## Notes

**Cite this article as:** Rodrigues de Souza , M, Aparecida Côrtes, M, Carlos Lucena da Silva, G, Jorge Fontoura Solla, D, Garcia Marques, E, Luz Oliveira Junior, W, Ferreira Fagundes, C, Jacobsen Teixeira, M, Luis Oliveira de Amorim, R, Rubiano, AM, Kolias, AG, and Silva Paiva, W (2022) Evaluation of computed tomography scoring systems in the prediction of short-term mortality in traumatic brain injury patients from a low- to middle-income country. *Neurotrauma Reports* 3:1, 168–177, DOI:10.1089/neur.2021.0067.

## Abbreviations Used

AUC: area under the receiver operating characteristic curve
CT: computerized tomography
GCS: Glasgow Coma Scale
ICP: intracranial pressure
ICU: intensive care unit
IMPACT: International Mission for Prognosis and Clinical Trials in TBI
IVH: intraventricular hemorrhage
LMICs: low- to middle-income countries
ROC: receiver operating characteristic
TBI: traumatic brain injury
TRIPOD: Transparent Reporting of a multi-variable prediction model for Individual Prognosis or Diagnosis
tSAH: traumatic subarachnoid hemorrhage
