# Supplementary material for: Evaluation of Computed Tomography Scoring Systems in the Prediction of Short-Term Mortality in Traumatic Brain Injury Patients from a Low- to Middle-Income Country
Source: Neurotrauma Rep. 2022 Apr 14;3(1):168–77. doi: 10.1089/neur.2021.0067 (PMC9081064; doi:10.1089/neur.2021.0067)
Supplement: Supplemental data [file Suppl_TableS1.docx]

**S1 Table.** Comparison of ROC curves using the model by DeLong et al.

|  | Marshall  (AUC) | Rotterdam  (AUC) | Helsinki  (AUC) | p value |
| --- | --- | --- | --- | --- |
| 14-day mortlaity | .610  .610  - | .762  -  .762 | -  .752  .752 | < .001  < .001  .621 |
| In-hospital mortality | .575  .575  - | .712  -  .712 | -  .716  .716 | < .001  < .001  .818 |

AUC area under roc curve
