# Supplementary material for: Evaluation of Computed Tomography Scoring Systems in the Prediction of Short-Term Mortality in Traumatic Brain Injury Patients from a Low- to Middle-Income Country
Source: Neurotrauma Rep. 2022 Apr 14;3(1):168–77. doi: 10.1089/neur.2021.0067 (PMC9081064; doi:10.1089/neur.2021.0067)
Supplement: Supplemental data [file Suppl_TableS2.docx]

**S2 Table.** Variation explained (Nagelkerke´s pseudo R^2^) of base model and subcomponents CT scores

|  | ***p* value** | **Pseudo R^2^** |
| --- | --- | --- |
| **Rotterdam CT Class** |  |  |
| Basal cisterns | .000 | .215 |
| Midline shift | .000 | .087 |
| Epidural mass lesion | .028 | .016 |
| Intraventricular hemorrahage | .000 | .043 |
| tSAH | .000 | .048 |
| **Helsinki CT Class** |  |  |
| *Mass lesion type* |  |  |
| Subdural hematoma | .000 | .096 |
| Intracerebral hematoma | .110 | .009 |
| Epidural hematoma | .028 | .016 |
| Hematoma volume | .153 | .007 |
| Intraventricular hemorrahage | .000 | .043 |
| Suprasselar cisterns | .000 | .176 |
| **Base Components** |  |  |
| Age | .000 | .057 |
| Motor score | .000 | .157 |
| Pupil responsiveness | .000 | .224 |
| Hypoxia | .002 | .060 |
| Hypotension | .001 | .039 |
| Hemoglobin | .088 | .010 |

tSAH traumatic subarachnoid hemorrhage
